# Inequality in the household and rural–urban migration in Ethiopian farmers

**DOI:** 10.1017/ehs.2020.10

**Published:** 2020-04-24

**Authors:** Lucie Clech, James Holland Jones, Mhairi Gibson

**Affiliations:** 1 Department of Anthropology and Archaeology, University of Bristol, 43 Woodland Rd, Bristol BS81TH, UK; 2 Department of Anthropology, Stanford University, 50, 450 Serra Mall, Stanford, CA 94305, USA; 3 Department of Earth System Science, Stanford University, 473 Via Ortega, Stanford, CA 94305 USA

**Keywords:** labour migration, intra-household resource allocation, selectivity, parental investment

## Abstract

Parental investment theory predicts that biases in investment favour migration by driving some of the sibling group to disperse for resources. Here we test hypotheses arising from this theory to explain patterns of rural–urban migration in south-central Ethiopia considering familial and individual strategies. We focus on the migration of low-skilled men, predicting two scenarios based on a low level of resource availability. Firstly, last-born sons will be more likely to migrate in order to offset their intra-household disadvantage when resources are limited (sibling competition). Alternatively, in households facing livelihood insecurity, older sons will migrate in order to free resources for their younger dependant brothers (reflecting sibling cooperation). Demographic, economic and relational data were collected from 217 families of male migrants, including information for 830 male adults. We performed multivariate analyses, including Bayesian generalised linear models and mixed models, to analyse quantitative data with a focus on household and individual likelihood of out-migration. Consistent with the predictions from parental investment theory, migration is dependent on intra-household resource allocation. Depending on the stage of the family cycle and livelihood context, families and individuals present different strategies: labour migration may result from sibling competition or from cooperation for resource enhancement.

## Graphical abstract

**Figure:**
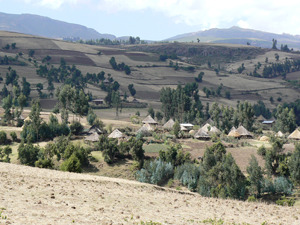

**Media summary:** Household's domestic cycle, sibling cooperation and competition help to explain labour migration in Ethiopian farmers

## Introduction

An important part of the literature on contemporary migration focuses on the selectivity of migration, as not all classes of individuals are equally likely to migrate (Bratti *et al.* 2016; Lall *et al.* 2006; Taylor 1987; VanWey 2005). Labour migration is reasonably interpreted as an adaptive strategy for the migrating individuals and/or for their kin, as migrants often support their kin through remittances (Corbett 1988; Hampshire 2002; Hampshire and Randall 1999; Konseiga 2007; McLeman and Smit 2006; Morrissey 2008). Labour migration can improve the embodied capital of migrants and their kin through social, material, human and biological capital (examples in de Brauw *et al.* 2018; de Brauw *et al.* 2011; de Brauw and Rozelle 2008; Hampshire 2002; Hampshire and Randall 1999; Hu 2012; Jampaklay 2006a, b; Katz and Stark 1986; Lancaster and Kaplan 2010; Li et *al.* 2007; Macours and Vakis 2010; McKenzie and Hildebrandt 2005; Quisumbing and McNiven 2010).

Despite having a direct link with the selectivity of migrants, the family configuration has been relatively understudied in social sciences up until recently (Bratti et *al.* 2016). However, evolutionary studies on past migration highlight the importance of intra-household resource access, through the analysis of family configurations and the competitive effects of siblings. For example, dynamic modelling of dispersal (Towner 1999) has shown the importance of environmental conditions, individual wealth access, inheritance and marriage probability in a land-based economy. Parental investment includes large wealth transfers, because these resources might affect the fitness of both parents and children (Hrdy and Judge 1993). As parental resources are limited, investment in a child is done at the expense of others; siblings are competing for these resources (Trivers 1974). Thus, parental investment, sibling competition and cooperation determine offspring access to resources (Borgerhoff Mulder 1998; Mock and Parker 1997; Trivers 1974), and might help in understanding out-migration for resources. For example, it has been found that sibling competition affected the likelihood of out-migration for the sons of farmers in eighteenth- and nineteenth-century rural Ireland (Strassmann and Clarke 1998), in eighteenth-century rural Sweden (Clarke and Low 1992), in the eighteenth- and nineteenth-century German Krummhörn population (Beise and Voland 2008; Voland and Dunbar 1995), in rural Massachusetts (Towner 2001), in preindustrial Finland (Nitsch *et al.* 2016) and in late nineteenth-century Colorado (Glover and Towner 2009).

Ethiopia offers a unique context for the study of rural–urban migration: it is the second most populous African country after Nigeria, one of the least urbanised (~20% of its population) and its urban population is expected to triple over the coming decades (2007–2037), more and more fuelled by rural–urban migration (Ozlu *et al.* 2015). In contemporary rural Ethiopia, large family sizes and land saturation are some of the factors explaining the impoverishment of the new generations (Nega et *al.* 2003). Rural families are challenged in their inheritance decisions and face a dilemma: partitioning their land among their offspring leaves subsequent generations poorer than the present one but maintaining the socio-economic status of the lineage typically requires favouring some offspring at the expense of others (Hrdy and Judge 1993). Despite a law dictating equal rights to inheritance, older sons are more likely to inherit land or better-quality land from their families (Congdon Fors *et al.* 2019; Gibson and Gurmu 2011). Fathers transfer land to their sons for, or prior to, their marriage, in order to allow them to start and sustain their separate household (Fafchamps and Quisumbing 2002, 2005a, b). Not surprisingly, Fafchamps and Quisimbung (2002) found a competitive effect of the number of brothers on inheritance. Gibson and Gurmu (2011) have shown that later-born farmers were disadvantaged in terms of agricultural productivity, marriage and reproductive success when land was inherited while Bezu and Holden (2014) found that later-born sons were more likely to engage in off-farm wage employment because they were less likely to inherit and to have access to land. Finally, Gibson and Gurmu (2012) found sibling competition to push out some household members as the birth of a younger sibling doubled the odds of out-migration.

Here we use an evolutionary framework to understand contemporary, rural–urban, male labour migration in the early stage of urbanisation, with a focus on intra-household access to resources. We argue that an evolutionary approach can improve understanding of how and why people vary in migration strategy. This paper focuses on trying to understand why, in migrants’ families, some sons are out-migrating for low-skilled employment in town, while others are not. We focused on families with at least three sons from the same father in order to test brothers’ competition and cooperation inside the same patriline. Depending on the household livelihood context, low-skilled labour migration might be an offsetting strategy for the disadvantaged offspring in the intra-household resource competition (Beise and Voland 2008; Towner 2002; Strassmann and Clarke 1998; Clarke and Low 1992), but also could be a cooperative strategy to support their kin (Bowles and Posel 2005).

Thus, we predict two possible outcomes regarding the effect of sibling competition and cooperation on migration. First, last-born sons are more likely to migrate for their first migration as low-skilled workers, compared with their brothers. Indeed, they are disadvantaged for intra-household resource access, such as land access owing to a multigeniture effect (multiple heirs) with first-born advantage (Gibson and Gurmu 2011). Second, alternatively, migration of sons might be a coping strategy for the household when dealing with unexpected circumstances affecting their livelihood security (Ezra and Kiros 2001). When last-born sons are too young to migrate, families might send away first- and/or middle-born sons who will act as cooperators for resource enhancement, freeing resources for the younger sons and/or trying to provide remittances (Bowles and Posel 2005), in other words being cooperative breeders (Rende Taylor 2004).

## Methods

### Local context

Following the 1978 Ethiopian Marxist revolution, the government confiscated and redistributed farm land periodically until the early 1990s. Each family received land of equal agricultural value, according to the number of people in the family (Rahmato 1985). Since then, land remains the property of the government; only land rights were distributed to farmers, who can pass them on to their offspring. In the study population, marriages are mostly patrilocal. Polygyny occurs for a few Muslims families (Gibson and Mace 2007; Fafchamps and Quisumbing 2002). Separations and divorces are possible for the Christians and are not frequent for the Muslims. Bride price and dowries are practised but account for a small proportion of the transfers that take place at the time of the marriage (Fafchamps and Quisumbing 2002).

### Data collection

Adama, the capital city of the Oromia region, was chosen over other urban centres because of its large migrant population and its proximity to rural communities previously studied (Gibson and Gurmu 2012). We collected data about low-skilled migrants, their natal household and their siblings through interviews of migrants in migrant-frequented spots. Labour migrants, while neither especially rare nor stigmatised, present many of the challenges faced with sampling hidden populations (Watters and Biernacki 1989). The informality of the labour and the fluidity and impermanence of their living arrangements mean that there is no straightforward sampling frame. We employed a targeted sampling strategy of visiting locations that informal day-labourers are visiting daily such as local job agencies for low-skilled workers, dormitories, restaurants, shops and even streets in a central district of the city known for its migrant population. The vast majority agreed to be interviewed (>90% of the respondents). Our targeted sampling strategy makes generalisation from the sample problematic, but allowed us to actually acquire a sample.

Interviews and questionnaires, following standard protocols for anthropological data collection (Bernard 2002; Fowler 1995), were undertaken in 2009. The data were collected with mixed methods: questionnaires completed during interviews, focus-group discussions and semi-directed qualitative interviews in rural and urban areas (Tashakkori and Teddlie 2003). Five-hundred and twenty-two migrants were interviewed about themselves, their families and their siblings. From this, only rural families from male respondents, with parents involved in farming activities, were kept in order to focus on rural–urban migration. Only families of respondents who first migrated for labour reasons after 14 years old were considered. Some of them had migrated several times. Families of respondents who first migrated for marriage, to follow their family, for education, for military service or for child labour migration (under 15) were not included. Both permanent and non-permanent (temporary or seasonal) migrations were included in labour migration. Families with stepfathers were removed in order to avoid complex family configurations, biases owing to reconfiguration of the families and complex inheritance strategies.

### Statistical analysis

A study such as this presents analytical challenges because many families have multiple sons in the analysis, potentially biasing results because of intra-family correlations. We therefore used Bayesian generalised mixed linear models (BGLMM) and Bayesian generalised linear models (BGLM), both with a logistic link function, implemented in the brms package in R (R version 3.6.1) to assess the impact of a number of factors on the probability of out-migration. Important covariates included in the models fall into three broad catergories: (1) family configuration; (2) family status and father; and (3) land access. Characteristics about family configuration included adult male sibship size (>14 years old), number of under-aged males (<15 years old), intra-sex birth-order category (first, middle or last-born sons). Information about family status and father variables included parental farmland size (current or at the death of the father), if their father was alive and years since the birth of their father (as a proxy for intergenerational age difference). Variables about land access included individuals’ current land size and extra land size expected from land transfer/inheritance in the future (estimation provided by the respondent for all sons). All models also included the current age of the individuals. For all models, we also tested the following interactions: birth-order categories × land size; birth-order categories × male sibling size; land size × male sibling size, parental land size × male sibling size. We performed model selection via Bayes factors, following the heuristic scale of Lee and Wagenmakers (2013). Families (family ID number) were specified as a random factor to control for their associated intra-family correlation. Data about the expected duration of the current migration (permanent/non-permanent), if remittances were sent in the past year, the distance of current migration from the birthplace, ethnicity and religion were also collected and are presented in the Appendix.

### Sample description

For the multivariate analyses, only families with more than two sons were considered, including 217 interviewees and their male sibling, for a total of 830 male individuals. The 290 labour-migrants were young (median = 22) and 85.2% were landless. Labour migrants were significantly more likely to be landless, to have received less than 0.5 ha, and to expect a future land size smaller than 0.5 ha compared with their brothers (Table 3). Of the 217 families, only four were landless and therefore unable to transfer land to the next generation. Most of the 217 families (60.4%) had 2 ha or more of land to transfer to their descendants (40.1% had 3 ha or more). Of the 217 respondents, only 19.4% had sent remittances in the past year. Of the 103 individuals who migrated more than a year ago, 27.2% had sent remittances to their family in the past year.

## Results

We employed both BGLMM and BGLM to test the effect of birth-order categories, family configuration and land transfers on the selected sample (*n =* 830) and then on two subsamples of families: families with adult-only sons (*n =* 517) and families with adult and under-aged sons (*n =* 313).

### Families with three sons or more: last-born sons are more likely to migrate

**Figure 1.**
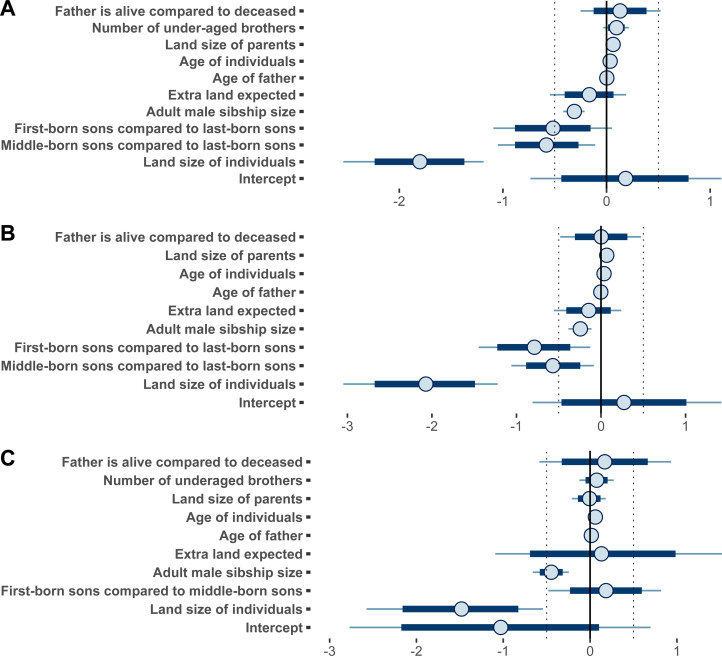
Posterior means and 80% and 95% density intervals. Details about the models are presented in Table 1. (a) Bayesian generalised linear models (BGLM) 1 (*n* = 830), model 1 in Table 1; (b) BGLM 2 (*n =* 517, sons from families with adult sons only), model 2 in Table 1; (c) BGLM 3 (*n =* 313, sons from families with under-aged sons), model 3 in Table 1

Analyses were performed on 217 families, including 830 adult sons of farmers to test the effect of intra-sex male birth-order categories. Of these men, 290 were rural–urban labour migrants; their first migration was for employment. A BGLMM, with a logistic link function for the probability of labour migration was used while controlling for family level (family ID number) as a random factor. This analysis revealed a small effect of the family-level factor (estimate = 0.11, 95% CI [0.00, 0.30]). When a BGLM was compared with the BGLMM, the Bayes factor of BF10 = 43.90, suggests that there is very strong evidence in favour of the BGLM (the non-mixed model; Table 1, model 1 and Figure 1a).

**Table 1.** Bayesian generalised linear models (BGLM) with a logistic link function, for the likelihood of labour migration

	Model 1: *n =* 830 families of 3 or more sons			Model 2: *n =* 517 sibships with adult sons only, families of 3 or more sons			Model 3: *n =* 313 sibships with adult and under-aged sons, families of 3 or more sons		
Variables	Estimates (+SE)	Lower 95% CI	Upper 95% CI	Estimates (+SE)	Lower 95% CI	Upper 95% CI	Estimates (+SE)	Lower 95% CI	Upper 95% CI
Intercept	0.17 (0.47)	−0.72	1.10	0.27 (0.58)	−0.86	1.36	−1.05 (0.87)	−2.83	0.62
Age of individuals (years)	0.03 (0.01)	0.01	0.06	0.04 (0.02)	0.01	0.07	0.06 (0.03)	0.00	0.12
Number of under-aged younger brothers	0.10 (0.06)	−0.02	0.22				0.08 (0.10)	−0.11	0.26
Adult male sibship sizes	−0.31 (0.05)	−0.41	−0.21	−0.25 (0.07)	−0.39	−0.11	−0.44 (0.10)	−0.65	−0.25
Middle-born sons/last-born sons	−0.57 (0.23)	−1.02	−0.12	−0.657 (0.26)	−1.10	−0.07			
First-born sons/last-born sons	−0.50 (0.29)	−1.07	0.05	−0.79 (0.34)	−1.47	−0.12			
First-born sons/middle-born sons							0.18 (0.33)	−0.46	0.85
Parental land sizes (current or at death of father, ha)	0.06 (0.03)	0.00	0.14	0.07 (0.04)	−0.00	0.15	−0.01 (0.10)	−0.21	0.19
Current land sizes of the individuals (ha)	−1.81 (0.34)	−2.51	−1.19	−2.08 (0.46)	−3.05	−1.23	−1.45 (0.51)	−2.56	−0.53
Extra land sizes expected from family in the future (ha)	−0.17 (0.19)	−0.56	0.18	−0.14 (0.20)	−0.56	0.23	0.15 (0.66)	−1.14	1.49
Father was alive/dead	0.13 (0.19)	−0.24	0.51	0.00 (0.23)	−0.46	0.45	0.19 (0.37)	−0.54	0.94
Number of years since the birth of father	0.00 (0.01)	−0.01	0.02	−0.00 (0.01)	−0.02	0.01	0.01 (0.01)	−0.01	0.04

An effect of birth-order categories was noticeable in the BGLM: first- and middle-born sons were credibly less likely to migrate compared with last-born sons (first/last estimate: −0.50, 95% CI [−1.07, 0.05], middle/last estimate: −0.57, 95% CI [−1.02, −0.12], model 1, Table 1). Compared with another reduced model (that is, a model containing no birth-order categories), this model (with birth-order categories) was more credible given the data (BF10 = 5.75, moderate evidence).

The analysis revealed an effect of family composition in the BGLM. Adult male sibship size presented a negative effect (estimate: −0.31, 95% CI [−0.41, −0.21]) while the under-aged male sibship size present a positive effect (estimate: 0.10, 95% CI [−0.02, 0.22]). In order to understand exactly how the family composition impacted the likelihood of labour migration, the sample was split into two subsamples: one of families with only adult sons (model 2, Table 1) and one of families with both adult and under-aged sons (<15 years old, model 3, Table 1).

Both subsamples were similar in terms of frequencies of landless sons, families with multiple labour migrants, parental land size, individual current and expected land size and remittances (Tables 2 and 3). Labour migrants from families with under-aged sons were significantly younger (>4 years difference), as were their fathers (~9 years difference), who were also significantly more likely to be alive compared with the fathers in families with adult sibships (Tables 2 and 3). Migrants from adult sibships had been migrating for a longer time than migrants from families with under-aged sons. Respondents from families with under-aged sons migrated at a younger age than respondents with adult sibships. Finally, when asked about the expected duration of their migration, labour migrants from adult sibships were significantly more likely to be reported by the respondent as permanent migrants compared with labour migrants from adult-and-juvenile sibships. This suggests different strategies from those individuals and families (Table 3).

**Table 2.** Inter-group comparison for the families with adult-only sibships and with adult-and-juvenile sibships (*t*-test for independence)

	Adult sibships			Adult and juvenile sibships						95% CI	
	Mean	SD	*n*	Mean	SD	*n*	d.f.	*t*	Significance	Lower	Upper
*Labour migrants comparison*											
Land sizes for labour migrants	0.07	0.21	167	0.08	0.40	123	172.74	−0.15	0.879	−0.08	0.07
Extra land sizes expected for labour migrants	0.38	0.59	167	0.33	0.42	123	287.61	0.93	0.354	−0.06	0.17
Age of labour migrants	26.37	8.17	167	21.93	7.09	123	280.25	4.94	*p* < 0.0001	2.67	6.21
*Respondent comparison*											
Years since first migration	2.14	3.34	119	1.14	1.94	98	194.77	2.75	0.006	0.28	1.72
Age of respondents at first migration	22.74	7.26	119	19.19	3.46	98	178.83	4.72	*p* < 0.0001	2.06	5.03
*Families comparison*											
Age of fathers	63.74	17.23	119	54.99	13.01	98	213.47	4.26	*p* < 0.0001	4.70	12.80
Parental land size	2.82	3.49	119	2.79	2.91	98	214.96	0.07	0.942	−0.82	0.89

**Table 3.** Inter-group comparisons for labour migrants/non-labour migrants and adult-only sibships/adult-and-juvenile sibships (Pearson *χ*^2^ tests for independence with Yates’ continuity correction)

	Labour migrants	Other brothers					
	%	*n*	%	*n*	d.f.	*χ*^2^	Significance
Landless individuals	14.8	290	60.4	540	1	156.64	<0.0001
Individuals with less than 0.5 ha	94.5	290	77.8	540	1	37.22	<0.0001
Individuals with future land size (current + expected) inferior to 0.5 ha	74.1	290	62.0	540	1	11.82	0.0006
	Adult sibships		Adult and juvenile sibships				
	%	*n*	%	*n*	d.f.	*χ*^2^	Significance
Individuals have not received land	67.3	517	71.9	313	1	1.70	0.192
Families have multiple labour migrants	25.2	119	19.04	98	1	0.74	0.391
Families have a father alive	64.7	119	86.7	98	1	12.64	0.0004
Respondents have not sent remittances in the past year (families with 2 sons or more)	75.6	119	86.7	98	1	3.56	0.059
Labour migrants were reported as permanent migrants/temporary migrants	74.3	167	61.8	123	1	4.57	0.032

### Adult-sons-only families: last-born sons are more likely to migrate

Similar analyses (BGLMM and BGLM) were performed on 517 men from 119 families with only adult sons (15+ years old). A total of 167 were reported to be rural–urban labour migrants. Parents, according to their landholding size, were probably of medium wealth or in the wealthier half of their community (median *=* 2). Again, a model without random factor (BGLM) was preferred to the one (BGLMM) including family level (BF10 = 43.70, very strong evidence; Table 1: model 2, Figure 1b).

In the BGLM, first- and middle-born sons were credibly less likely to migrate compared with last-born sons (first/last estimate: −0.79, 95% CI [−1.47, −0.12], middle/last estimate:−0.57, 95% CI [−1.10, −0.07], model 2, Table 1). In comparison with a reduced model containing no birth-order categories, the model with birth-order categories was more credible given the data (BF10 = 11.31, strong evidence).

In the BGLM, current land size of the respondent had a negative effect on migration (estimate: −2.08, 95% CI [−3.05, −1.23]), and the model (including land size) is more credible compared with a reduced model without land size (BF10 = 18,510,492.21, extreme evidence). Extra land expected in the future and competition with their father for land access seemed not to be of importance for dispersal in these models, as neither intergenerational age difference (number of years since the birth of father/age of father) nor if the father was deceased/alive presented an effect (Figure 1b).

Finally, parental land size presented a positive effect (estimate: 0.07, 95% CI [0.00, 0.15]), suggesting that families with large land sizes were more likely to have more than one migrant compared with families with smaller land. Results also indicates that the number of brothers (>14 years old) had a negative effect (estimate: −0.25, 95% CI [−0.39, −0.11]), suggesting that a large male sibship size was less likely to have several migrants compared with a small sibship.

### Adult-and-juvenile-sons families: first- and middle-born sons are more likely to migrate

Similar analyses (BGLMM and BGLM) were performed on 313 men from 98 families with both adult and under-aged sons. A total of 123 were reported to be rural–urban labour migrants. Again, parents, according to their landholding size, were probably of medium wealth or in the wealthier half of their community (median *=* 2). Again, the BGLM was preferred to the BGLMM (BF10 = 57.62, very strong evidence; Table 1, model 3, Figure 1c).

In this subsample, first- and middle-born sons were the only ones migrating; last-born sons were under-aged for adult labour migration. Results in the BGLM suggest that birth-order categories have no effect; first-born sons were migrating neither more nor less than middle-born sons (first/middle estimate: 0.18, 95% CI [−0.46, 0.85]. Compared with a reduced model (that is, a model containing no birth-order categories), the Bayes factor = 0.96, suggesting that neither model is preferred given the data.

In the BGLM, the current land size of the respondent had a negative effect on migration (estimate: −1.45, 95% CI [−2.56, −0.53]), and the model (including land size) is more credible compared with a reduced model, without land size (BF10 = 209.13, extreme evidence). Parental land size and extra land expected did not present any effects on migration (Figure 1c). Again, competition with their father for land access seemed not to be of importance for dispersal, as neither intergenerational age difference (number of years since the birth of father/age of father) nor if the father was deceased/alive presented an effect (Figure 1c).

Finally, the results suggest that the number of brothers (>14 years old) had a negative effect (estimate: −0.44, 95% CI [−0.65, −0.25]) suggesting that families with more adult sons were less likely to have multiple labour migrants compared with families with fewer adult sons. Results also suggest that the number of under-aged brothers (<15 years old) had almost no impact on the likelihood of migration (estimate: 0.08, 95% CI [−0.11, 0.26]).

## Discussion

Using parental investment (PI) theory to generate hypotheses in this study, we have tested the role of intra-household resource access in driving the decision of low-skilled men to engage in labour migration. Previous evolutionary studies were based on historical records. In contrast, our study is based on ethnographic data specifically gathered on contemporary labour migration in south-central Ethiopia to test the hypotheses drawn from PI theory. We use a mixed-method approach to control for potentially confounding effects of intra-family correlation and focus on the destination of rural–urban labour migration. Past studies have shown that some individuals migrate because they are disadvantaged in the sibling competition for resources or mate access (Beise and Voland 2008; Clarke and Low 1992; Strassmann and Clarke 1998; Towner 2001; Voland and Dunbar 1995). Other studies present migrants as cooperators because they support their kin through remittances (Azam and Gubert 2006; Bowles and Posel 2005; De Brauw *et al.* 2011). Here, in this contemporary rural population, we found that both are happening, depending on the livelihood context and stage in the household's domestic cycle. We identified two patterns of out-migration, with opposing effects of birth order. In the first case, last-born individuals migrate owing to an intra-household disadvantage in terms of resource access. In the latter case, eldest sons (specifically the eldest unmarried sons) migrate to free resources for the younger sons and/or in the hope of bringing remittances.

### Families with three sons or more: last-born sons are more likely to migrate

Migrants in our sample were mostly young, landless, unmarried and childless, confirming other findings in Ethiopia (Kosec *et al.* 2017; Gibson and Gurmu 2012; Ezra and Kiros 2001) and elsewhere (Lall *et al.* 2006; de Haan 1999). Our results revealed a birth-order effect on the likelihood of labour migration: last-born sons were more likely to migrate compared with their brothers. When taking into account family configuration, the number of adults and under-aged siblings appeared to have some importance, suggesting the necessity of analysing separately two groups of families, which represent different stages of families’ domestic cycles (Davis and Daly 1997; Emlen 1995; Fortes 1958). The first group includes sibships with only adult sons, who are theoretically in or near reproductive age, and have dispersed or will disperse soon from the natal household to start their own household. Not surprisingly, fathers were older and were less likely to be alive compared with the second group. The second group includes sibships with under-aged sons. In these, part of the sibship still needs to survive to reach reproductive age and is still dependent on family cohesion and cooperation for survival. When asked about the expected duration of the current migration, sons from adult sibships were more likely to have migrated at a later age and to be permanent migrant compared with sons from adult and under-aged sibships, supporting the idea that families and individuals engage in different strategies in those two groups.

### Families with only adult sons: last-born sons are more likely to migrate

The first subsample, comprising adult sibships, revealed a positive effect for being last-born on the probability of out-migration. Previous work in a similar community, focusing on the number of older brothers, has shown that later-born farmers were disadvantaged in agricultural productivity, marriage and reproductive success (Gibson and Gurmu 2011). Another study in a similar rural community has shown that last-born settled farmers have a significantly smaller kin network but tend to have an extended non-kin network compared with their brothers (Clech *et al.* in press). In addition, last-born sons might enjoy a different type of social capital compared with their older brothers as they might accumulate knowledge through the social networks of their older siblings, which potentially reduces the cost of labour migration for them. In other words, last-born sons could have a combination of characteristics that disadvantage them in their home community, but that may benefit them during migration.

Studies of past rural western populations have shown that non-inheriting or less-favoured inheriting children might have a less promising future compared with their brothers and could face downward social mobility, remain single or migrate (Strassmann and Clarke 1998; Towner 2001; Voland and Dunbar 1995). Logically, difficulty in land access might delay marriage or create a disadvantage in finding a bride and for starting one's own household and subsequently impact reproductive life in general (Strassmann and Clarke 1998; Voland and Dunbar 1995). During focus groups in villages, elders reported that social norms favour class endogamy for marriage agreement, confirming other results (Fafchamps and Quisumbing 2002, 2005b). Numerous migrants reported not being able to settle down and start a family in their village. Other findings confirmed these results (Kosec *et al.* 2017; Bezu and Holden 2014). Generational impoverishment (Nega *et al.* 2003) appears to impact last-born sons more drastically than other sons and leaves them with poor marriage prospects in their community. The last-born effect is noticeable even when parental biases in terms of past and future land transfer were added into the models. Later-born sons might be disadvantaged in terms of land productivity, marriage, reproductive success (Gibson and Gurmu 2011) and inheritance (Bezu and Holden 2014), but among later born sons, last-born sons are the most likely to migrate out. For them, labour migration could be seen as an attempt to gain economic independence in order to be able to start their own households, and to transition to their next life-cycle stage, instead of remaining landless, single and dependent on their natal household.

### Families with adult and juvenile sons: first- and middle-born sons are more likely to migrate

The second subsample included sibships with both adult and under-aged males and revealed that first- and middle-born sons were more likely to emigrate. Labour migrants were on average four and a half years younger in this subsample compared with the other subsample, and more likely to be reported as temporary migrants. According to focus-group discussions in both rural and urban areas, family strategies could be mixed, depending on who is present in the household at any given moment. Logically, according to many elders, families send away the oldest son living with them at the time (i.e. the oldest who is still single and landless), because he will be the most mature and ready to find his way in the risky urban world. In other words, migration is more a family strategy than an individual strategy and appears to be a temporary solution to special circumstances affecting their livelihood security. However, those young migrants might benefit later from their urban experience and improve their value on the marriage market. For example, Jampaklay (2006a) has shown that individuals with migration experience are more likely to marry than those who have never moved out of their home villages in Thailand.

### Is migration driven by poverty and vulnerability?

The picture is more complicated. Many respondents indicated the lack of rain as the reason for the lack of resources and jobs in their original community. Viste *et al.* (2013) noted that rainfall was below average for several consecutive years, including the year data were collected (Viste et *al.* 2013). These results corroborate previous work conducted in Ethiopia; Gray and Mueller (2012) have shown that drought has non-negligible consequences for labour-related moves for men.

### Individual land access

In the context of generational impoverishment (Nega *et al.* 2003) our results show that land inheritance has a negative effect on migration, confirming the findings of Kosec *et al.* (2017) and Bezu and Holden (2014). A higher proportion of low-skilled migrants were disadvantaged for land access compared with their brothers. Current land size, more than future inheritances and parental land size or intergenerational age difference, has a credible effect in our models.

Men who receive land are able to get married and to start their separate household; land access might prevent their migration by buffering certain shocks affecting livelihood security (Gray and Mueller 2012). Moreover, when land tenure is not secure, land-rights owners might decide to stay in their communities to protect their rights. In theory, under the local administrative system, land-rights owners lose their land rights when they are registered in another district. In other words, having land might protect farmers from shocks, but also might stop them from migrating for fear of losing their land rights. For example, in China, migration duration is negatively associated with the level of land insecurity (de la Rupelle *et al.* 2008). The current land certification programme in Ethiopia, by securing land tenure, might impact migration strategies and increase migration rates. We could imagine more settled farmers engaging in temporary migration, for example.

According to elders, ‘movers are losing their right to land inheritance because they are away and cannot use the land’, suggesting that inheritance expectations might play a role in the decision to migrate. However, in our models, future inheritance, intergenerational age difference and parental land size did not reveal any credible or consistent effect, suggesting that, when resources are scarce, the current level of resources might outweigh uncertain resource expectations in migration decisions.

### Family background

Level of material resources of the original household was expected to affect the likelihood of labour migration, with low-skilled labour migrants coming predominantly from poor families (Gray and Mueller 2012). Our results show that a large proportion of migrants actually came from average or wealthier households, relative to rural standards of the area for this generation. More than half of the labour migrants (59.4%) came from households with two or more hectares, (40.6% with three or more). These results confirm previous findings (Gibson and Gurmu 2012), suggesting a complicated wealth effect and different costs of migration according to socio-economic status. The economic costs of migration to Adama are not high, even from a rural perspective, making migration theoretically possible for all. Many migrants moved to the city with almost nothing, negotiating their transportation with truck drivers or in other ways, and finding very cheap accommodation, free informal shelter or, for the most unfortunate, sleeping on the streets on their arrival. Focus-group discussions in the rural area revealed that low-status households might adopt other coping strategies, suggesting different non-monetary and opportunity costs for the very poor compared with the less unfortunate. For example, an informant pointed out the importance of social and human capital such as child labour or child placement in other families: ‘Poor/low status people, give their child to others, not only to their relatives, but also to their neighbours. They grow up in other families as servants. The poor need to know somebody in the city to send their child to and most do not.’

Hampshire (2002) has also shown that short-term labour migration among Fulani in Burkina Faso was not a response to destitution but rather a way to enhance livelihood security for wealthier households. In our study, the costs of migration and remittance rates were low, confirming other findings on internal migration in Ethiopia (De Brauw *et al.* 2011) and suggesting the possible importance of perceived future remittances (vs reality) and other benefits of migration for the household. In families with under-aged sons, migrants often reported cooperating for household resource enhancement because they were immediately freeing resources for the younger siblings.

Our models reveal some inter-household differences. In all models, families with fewer adult sons (but conditional on having at least three sons) were more likely to have multiple labour migrants compared with families with many sons. Numerous adult brothers might act as a safety net for the lineage, limiting the need for migration of several unmarried brothers, or reflect the higher status of the family, which might engage in other types of livelihood strategies. Families with few brothers might also suggest more vulnerable households, particularly if the small number of sons arises from high mortality.

As previous results on wealth suggested, parental land size did not have a consistent effect across models, and revealed only a small positive effect for families with adult male sibships.

Finally, families with a deceased father or with a younger father were not more likely to have more migrants compared with others. Because we targeted migrants in the destination area, our sample includes households with at least one migrant. As such, we are unable to discriminate differences between the different household classes and the general rural population. More general statements about the role of labour migration in rural livelihood strategies would require a broader, more fully representative sample of rural households. We are further limited in our ability to make general statements by the necessity of employing targeted sampling for this migrant population lacking a sampling frame.

## Conclusion

As expected, resource context matters. Low-skilled labour migration may be the result of an intra-household resource competition or it may be a cooperative strategy for household provisioning. However, our results show a more complex picture than our predictions. Both outcomes represent different stages of the family cycle (Fortes 1958), where parents are involved heavily in the decision of their son's migration. In families with only adult sons, last-born sons appear to be the less favoured in their families for indivisible large wealth transfers. For these individuals, migration is mostly a resource strategy for avoiding downward social mobility and should be considered in a broader picture including competition for finding a bride. Alternatively, but not exclusively, in families with juvenile sons, the oldest sons present in their household may be the best equipped to face the difficulties of city life when a shock (such as drought or crop failure) impacts their household livelihood security. Thus, by freeing resources and attempting to gather extra resources for their household and younger siblings, they act as remote ‘helpers-at-the-nest’ (Rende Taylor 2004). In these cases, resource status and the stage of family cycle appear to have an impact on those strategies. These patterns might be even more complex when controlling for other types of family structures that possibly impact parental investment and inheritance (e.g. families with stepfathers, mother and father who married multiple times, polygyny) and when including other types of migration. Our results suggest a switch in strategies following the age structure of the siblings. Further studies on the transitions with regards to favouritism are needed to understand if the change occurs once the youngest reaches 15, independence or marriage. This switch might reveal a trade-off in parental investment in survival vs. future reproductive status of their sons. Further work should focus on how and why this happens, in order to understand why households vary over space (e.g. between one another) and over time (e.g. transition from one another). From our data, the relationship between poverty and migration is not straightforward, as the very poor might engage in other types of coping strategies owing to their different resources and norms and their socio-economic positions (Hampshire 2002; Hampshire and Randall 1999). In all cases, low-skilled male labour migration should not be considered only as an optimal resource strategy but also needs to take into account the reproduction, livelihood security and social mobility of the individuals and their kin. A focus on the real socio-economic and demographic costs and benefits of migration is important for understanding who benefits from migration in terms of fitness (Voland and Dunbar 1995) and what trends in migration may emerge in the future (Gibson and Gurmu 2012).

## Supplementary material

For supplementary material accompanying this paper visit http://dx.doi.org/10.1017/ehs.2020.10. click here to view supplementary material

## Data accessibility

The datasets supporting this article have been uploaded as part of the Electronic Supplementary Material. The R code that supports the findings of this study is available from the authors on request.

## References

[ref1] Azam J-P and Gubert F (2006) Migrants’ remittances and the household in Africa: a review of evidence. Journal of African Economies 15(suppl 2), 426–462. 10.1093/jae/ejl030

[ref2] Beise J and Voland E (2008) Intrafamilial resource competition and mate competition shaped social-group-specific natal dispersal in the 18th and 19th century Krummhörn population. American Journal of Human Biology 20(3), 325–336. 10.1002/ajhb.20730 18186514

[ref3] Bernard H (2002) Research Methods in Anthropology: Qualitative and Quantitative Approaches. Lanham, MD: Altamira Press.

[ref4] Bezu S and Holden S (2014) Are rural youth in Ethiopia abandoning agriculture? World Development 64, 259–272. 10.1016/j.worlddev.2014.06.013

[ref5] Borgerhoff Mulder M (1998) Brothers and sisters. Human Nature 9(2), 119–161. 10.1007/s12110-998-1001-6 26197443

[ref6] Bowles S and Posel D (2005) Genetic relatedness predicts South African migrant workers’ remittances to their families. Nature 434(7031), 380–383. 10.1038/nature03420 15772661

[ref7] Bratti M, Fiore S and Mendola M (2016) *Family Size, Sibling Rivalry and Migration: Evidence from Mexico* (No. 390). Centro Studi Luca d'Agliano, University of Milano. Retrieved from: https://ideas.repec.org/p/csl/devewp/390.html

[ref8] Clarke AL and Low BS (1992) Ecological correlates of human dispersal in 19th century Sweden. Animal Behaviour 44(4), 677–693. 10.1016/S0003-3472(05)80295-7

[ref9] Clech L, Hazel A and Gibson M (2019) Does kin-selection theory help to explain support networks among farmers in south-central Ethiopia? Human Nature: An Interdisciplinary Biosocial Perspective 30, 422–447. 10.1007/s12110-019-09352-6 31729694

[ref10] Congdon Fors H, Houngbedji K and Lindskog A (2019) Land certification and schooling in rural Ethiopia. World Development 115, 190–208. 10.1016/j.worlddev.2018.11.008

[ref11] Corbett J (1988) Famine and household coping strategies. World Development 16(9), 1099–1112. 10.1016/0305-750X(88)90112-X

[ref12] Davis JN and Daly M (1997) Evolutionary theory and the human family. The Quarterly Review of Biology, 72(4), 407–435. 10.1086/419953 9407672

[ref13] de Brauw A and Rozelle S (2008) Migration and household investment in rural China. China Economic Review 19(2), 320–335. 10.1016/j.chieco.2006.10.004

[ref14] de Brauw A, Mueller V and Woldehanna T (2011) *Insurance Motives to Remit: Evidence from a Matched Sample of Ethiopian Internal Migrants* (ESSP II Working Paper 25). http://www.ifpri.org/publication/insurance-motives-remit-0?print

[ref15] de Brauw A, Mueller V and Woldehanna T (2018) Does internal migration improve overall well-being in Ethiopia? Journal of African Economies 27(3), 347–365. 10.1093/jae/ejx026

[ref16] de Haan A (1999) Livelihoods and poverty: the role of migration – a critical review of the migration literature. Journal of Development Studies 36(2), 1–47. 10.1080/00220389908422619

[ref17] de la Rupelle M, Quheng D, Shi L and Vendryes T (2008) Land rights and rural–urban migration in China. China Perspectives 2, 25–35.

[ref18] Emlen ST (1995) An evolutionary theory of the family. Proceedings of the National Academy of Sciences 92(18), 8092. 10.1073/pnas.92.18.8092 PMC41102 7667250

[ref19] Ezra M and Kiros G-E (2001) Rural out-migration in the drought prone areas of Ethiopia: a multilevel analysis. The International Migration Review 35(3), 749–771. JSTOR. 19186413 10.1111/j.1747-7379.2001.tb00039.x

[ref20] Fafchamps M and Quisumbing AR (2002) Control and ownership of assets within rural Ethiopian households. Journal of Development Studies 38(6), 47. bth.

[ref21] Fafchamps M and Quisumbing A (2002) *Marriage and Assortative Matching in Rural Ethiopia*, CSAE working papers series 2002-21, Centre for the Studies of African Economies, University of Oxford.

[ref22] Fafchamps and Quisumbing A (2005a) Assets at marriage in rural Ethiopia. Journal of Development Economics 77(1), 1–25. 10.1016/j.jdeveco.2004.02.003

[ref23] Fafchamps M and Quisumbing AR (2005b) Marriage, bequest, and assortative matching in rural Ethiopia. Economic Development and Cultural Change 53(2), 347–380. 10.1086/425373

[ref24] Fortes M (1958) Introduction. In J. Goody, The Developmental Cycle of Domestic Groups (p. 145). Cambridge: Cambridge University Press. Retrieved from: http://www.zoran-cuckovic.from.hr/materials/Goody-Developemental-Cycles.pdf

[ref25] Fowler F (1995) Improving Survey Questions: Design and Evaluation. Thousand Oaks, CA: Sage.

[ref26] Gibson M and Gurmu E (2011) Land inheritance establishes sibling competition for marriage and reproduction in rural Ethiopia. Proceedings of the National Academy of Sciences 108(6), 2200–2204. 10.1073/pnas.1010241108 PMC3038728 21262826

[ref27] Gibson M and Gurmu E (2012) Rural to urban migration is an unforeseen impact of development intervention in Ethiopia. Plos One 7(11), 1–8. 10.1371/journal.pone.0048708 PMC3498254 23155400

[ref28] Gibson M and Mace R (2007) Polygyny, reproductive success and child health in rural Ethiopia: why marry a married man? Journal of Biosocial Science 39, 287–300. 10.1017/S0021932006001441 16817989

[ref29] Glover S and Towner M (2009) Long-distance dispersal to the mining frontier in late 19th century Colorado. Behaviour 146, 677–700. 10.1163/156853908X395558

[ref30] Gray C and Mueller V (2012) Drought and population mobility in rural Ethiopia. World Development 40(1), 134–145. 10.1016/j.worlddev.2011.05.023 22523447 PMC3328858

[ref31] Hampshire K (2002) Fulani on the move: seasonal economic migration in the Sahel as a social process. Journal of Development Studies 38(5), 15–36. 10.1080/00220380412331322491

[ref32] Hampshire K and Randall S (1999) Seasonal labour migration strategies in the Sahel: Coping with poverty or optimising security? International Journal of Population Geography 5(5), 367–385. 10.1002/(sici)1099-1220(199909/10)5:5<367::aid-ijpg154>3.0.co;2-o 12349428

[ref33] Hrdy SB and Judge DS (1993) Darwin and the puzzle of primogeniture: An essay on biases in parental investment after death. Human Nature (Hawthorne, N.Y.) 4(1), 1–45. 10.1007/BF02734088 24214292

[ref34] Hu F (2012) Migration, remittances, and children's high school attendance: The case of rural China. International Journal of Educational Development 32(3), 401–411. 10.1016/j.ijedudev.2011.08.001

[ref35] Jampaklay A (2006a) How does leaving home affect marital timing? An event-history analysis of migration and marriage in Nang Rong, Thailand. Demography 43(4), 711–725. 10.1353/dem.2006.0035 17236543

[ref36] Jampaklay A (2006b) Parental absence and children's school enrolment. Asian Population Studies 2(1), 93–110. 10.1080/17441730600700598

[ref37] Katz E and Stark O (1986) Labor migration and risk aversion in less developed countries. Journal of Labor Economics 4(1), 134–149. 12267743 10.1086/298097

[ref38] Konseiga A (2007) Household migration decisions as survival strategy: the case of Burkina Faso. Journal of African Economies 16(2), 198–233. 10.1093/jae/ejl025

[ref39] Kosec K, Ghebru H, Holtemeyer B, Mueller V and Schmidt E (2017) The effect of land access on youth employment and migration decisions: evidence from rural Ethiopia. American Journal of Agricultural Economics 100(3), 931–954. 10.1093/ajae/aax087

[ref40] Lall SV, Selod H and Shalizi Z (2006) *Rural–Urban Migration in Developing Countries: A Survey of Theoretical Predictions and Empirical Findings* (3915). Retrieved from: http://ssrn.com/abstract=920498

[ref41] Lancaster J and Kaplan H (2010) Embodied capital and extra-somatic wealth in human evolution and human history. In MP Muehlenbein (ed). Human Evolutionary Biology (pp. 439–455). Cambridge: Cambridge University Press.

[ref42] Lee MD and Wagenmakers E-J (2013) Bayesian Cognitive Modeling: A Practical Course (Vol. 1-1 online resource (xiii, 264 pages): illustrations). Cambridge: Cambridge University Press. 10.1017/CBO9781139087759

[ref43] Li L, Wang H, Ye X, Jiang M, Lou Q and Hesketh T (2007) The mental health status of Chinese rural–urban migrant workers. Social Psychiatry and Psychiatric Epidemiology 42(9), 716–722. 10.1007/s00127-007-0221-0 17598056

[ref44] Macours K and Vakis R (2010) Seasonal migration and early childhood development. World Development 38(6), 857–869. 10.1016/j.worlddev.2010.02.012

[ref45] McKenzie D and Hildebrandt N (2005) *The Effects of Migration on Child Health in Mexico*. Retrieved from: http://papers.ssrn.com/sol3/papers.cfm?abstract_id=719041#

[ref46] McLeman R and Smit B (2006) *Migration as an Adaptation to Climate Change* (Vol. 76). 10.1007/s10584-005-9000-7

[ref47] Mock D and Parker G (1997) The Evolution of Sibling Rivalry. Oxford: Oxford University Press.

[ref48] Morrissey J (2008) Rural urban migration in Ethiopia. Forced Migration Review 28(31), 28–29.

[ref49] Nega B, Adenew B and Sellasie S G (2003) Current land policy issues in Ethiopia. Land Reform: Land Settlement and Co-Operatives (pp. 103–124). Rome: Food and Agriculture Organization.

[ref50] Nitsch A, Lummaa V and Faurie C (2016) Sibship effects on dispersal behaviour in a pre-industrial human population. Journal of Evolutionary Biology 29(10), 1986–1998. WorldCat.org. 10.1111/jeb.12922 27318237

[ref51] Ozlu MO, Alemayehu A, Mukim M, Lall SV, Kerr OT and Kaganova O (2015) *Ethiopia—Urbanization Review: Urban Institutions for a Middle-income Ethiopia*. World Bank Group. Retrieved from: http://documents.worldbank.org/curated/en/543201468000586809/Ethiopia-Urbanization-review-urban-institutions-for-a-middle-income-Ethiopia

[ref52] Quisumbing A and McNiven S (2010) Moving forward, looking back: the impact of migration and remittances on assets, consumption, and credit constraints in the rural Philippines. Journal of Development Studies 46(1), 91–113. 10.1080/00220380903197960

[ref53] Rahmato D (1985) *Agrarian Reform in Ethiopia*. Scandinavian Institute of African Studies, Uppsala. Red Sea Press. Retrieved from: http://www.diva-portal.org/smash/get/diva2:274402/FULLTEXT01.pdf

[ref54] Rende Taylor L (2004) Maintaining the matriline: children's birth order roles and educational attainment among Thai Khon Muang. In Socioeconomic Aspects of Human Behavioral Ecology (vol. 23, pp. 355–377). Bingley: Emerald. 10.1016/S0190-1281(04)23015-3

[ref55] Strassmann BI and Clarke AL (1998) Ecological constraints on marriage in rural Ireland. Evolution and Human Behavior 19(1), 33–55. 10.1016/s1090-5138(97)00103-7

[ref56] Tashakkori A and Teddlie C (2003) Handbook of Mixed Methods in Social & Behavioral Research. London: Sage.

[ref57] Taylor JE (1987) Undocumented Mexico–U.S. migration and the returns to households in rural Mexico. American Journal of Agricultural Economics 69(3), 626–638.

[ref58] Towner M (1999) A dynamic model of human dispersal in a land-based economy. Behavioral Ecology and Sociobiology 46, 82–94. 10.1007/s002650050596

[ref59] Towner M (2001) Linking dispersal and resources in humans. Human Nature 12(4), 321–349. 10.1007/s12110-001-1002-1 26192411

[ref60] Towner MC (2002) Linking dispersal and marriage in humans: life history data from Oakham, Massachusetts, USA (1750–1850). Evolution and Human Behavior 23(5), 337–357. 10.1016/s1090-5138(02)00094-6

[ref61] Trivers R (1974) Parent-Offspring Conflict. American Zoologist 14(1), 249–264.

[ref62] VanWey LK (2005) Land ownership as a determinant of international and internal migration in Mexico and internal migration in Thailand. International Migration Review 39(1), 141–172. 10.1111/j.1747-7379.2005.tb00258.x

[ref63] Viste E, Korecha D and Sorteberg A (2013) Recent drought and precipitation tendencies in Ethiopia. Theoretical and Applied Climatology 112(3), 535–551. 10.1007/s00704-012-0746-3

[ref64] Voland E and Dunbar RI (1995) Resource competition and reproduction: the relationship between economic and parental strategies in the Krummhorn population (1720–1874). Human Nature (Hawthorne, NY) 6(1), 33–49. 10.1007/BF02734134 24202829

[ref65] Watters JK and Biernacki P (1989) Targeted sampling: options for the study of hidden populations. Social Problems 36(4), 416–430. 10.1525/sp.1989.36.4.03a00070

